# Immediate Loading of 5.2-mm Ultra-Short Implants Supporting Maxillary Overdentures in the Severely Atrophic Maxilla: A Prospective Pilot Study

**DOI:** 10.3390/jcm15166250

**Published:** 2026-08-12

**Authors:** Pieter-Jan Boderé, Wout Scharff, Cyrille France, Stefan Vandeweghe, Hugo De Bruyn, Renaat Coopman, Luc Van Doorne

**Affiliations:** 1 Department of Reconstructive Dentistry, Faculty of Medicine and Health Sciences, Ghent University Hospital, Corneel Heymanslaan 10, 9000 Ghent, Belgium; 2 Department of Oral Health Sciences, Faculty of Medicine and Health Sciences, Ghent University Hospital, Corneel Heymanslaan 10, 9000 Ghent, Belgium; 3 Department of Oral and Craniomaxillofacial Surgery, Faculty of Medicine and Health Sciences, Ghent University Hospital, Corneel Heymanslaan 10, 9000 Ghent, Belgium; 4 Private Practice, Het Tandplein, Bilkske 68, 8000 Brugge, Belgium

**Keywords:** ultra-short implants, atrophic maxilla, implant-supported overdenture, immediate loading

## Abstract

**Background:** Severe maxillary atrophy often precludes placement of conventional dental implants and frequently requires invasive bone augmentation procedures. This prospective pilot study evaluated a treatment concept using immediately loaded 5.2-mm ultra-short implants connected by a patient-specific CAD/CAM inter-implant bar system (VD CLIC BAR^®^) to support maxillary overdentures. **Methods**: Thirteen completely edentulous patients with severe maxillary atrophy (Cawood–Howell Class V or VI) were treated with six to eight immediately loaded 5.2-mm ultra-short implants connected by a patient-specific CAD/CAM inter-implant bar (VD CLIC BAR^®^) supporting a maxillary overdenture. The primary outcome was implant survival. Secondary outcomes included oral health-related quality of life (OHIP-14), self-reported chewing ability (VAS), implant stability (Periotest), and prosthetic rehabilitation. **Results:** A total of 96 initial implants and 8 additional implants were placed following early implant failures. The primary implant survival rate after two years was 83.3%. Implant failures occurred predominantly during early healing and were unevenly distributed across the cohort. Early failures were managed by placement of additional ultra-short implants, enabling definitive implant-supported rehabilitation in all patients. Periotest values demonstrated a significant increase in implant stability over time (*p* < 0.001). Median OHIP-14 scores improved from 32.0 (IQR 20.0–44.0) at baseline to 4.0 (IQR 1.0–20.0) at two years (*p* = 0.006). Median chewing ability at two years was 82.7 (IQR 50.1–91.9). **Conclusions:** This prospective pilot study demonstrates the feasibility of an immediately loaded treatment concept using 5.2-mm ultra-short implants splinted with a patient-specific CAD/CAM inter-implant bar (VD CLIC BAR^®^) to support maxillary overdentures in patients with severe maxillary atrophy. Although early implant failures occurred and required placement of additional ultra-short implants in some patients, all patients ultimately achieved functional implant-supported rehabilitation. These preliminary findings require confirmation in larger controlled studies with longer follow-up.

## 1. Introduction

Severe maxillary atrophy remains one of the most challenging conditions in implant dentistry. Progressive alveolar bone resorption compromises the placement of standard-length implants and often leaves patients with unstable conventional dentures, resulting in impaired mastication, reduced oral function, and diminished oral health–related quality of life [1]. Implant-supported rehabilitations have substantially improved treatment outcomes in edentulous patients, but advanced bone loss frequently precludes the placement of conventional implants. In cases of advanced ridge atrophy, sufficient bone volume for conventional implant placement may require augmentation procedures such as maxillary sinus floor elevation, guided bone regeneration, onlay or interpositional grafting, or distraction osteogenesis. These procedures are well-established and can provide predictable outcomes, particularly when treating localized atrophic sites. However, in patients with severe generalized maxillary atrophy, they may require more extensive reconstruction, thereby increasing surgical complexity and substantially prolonging treatment. They may also be associated with donor-site morbidity, membrane perforation, graft failure, postoperative discomfort, and increased treatment costs [2,3].

To overcome these limitations, several graft-avoiding treatment concepts have been developed, including tilted, zygomatic, pterygoid, and patient-specific subperiosteal implants. These approaches have considerably expanded the treatment options for patients with severe maxillary atrophy and have demonstrated favorable clinical outcomes with high implant survival rates in appropriately selected patients. Nevertheless, they require advanced surgical expertise, meticulous treatment planning, and may be associated with procedure-specific complications. Moreover, when complications or implant failure occur, their management can be challenging and may require implant removal and additional surgical or prosthetic interventions [4,5,6,7,8,9]. Consequently, some edentulous patients remain insufficiently treated because of anatomical, medical, financial, or patient-related constraints [10].

Although these graft-avoiding approaches have expanded the available treatment options, they are not suitable for every patient. Consequently, interest has grown in less invasive treatment concepts that make optimal use of the available residual alveolar bone. Within this therapeutic spectrum, short and ultra-short implants may represent an intermediate treatment concept by utilizing the available residual alveolar bone and potentially reducing or avoiding the need for more extensive reconstructive or remote-anchorage procedures in carefully selected patients. Over the past decade, improvements in implant surface texture and implant design have led to the development of short and ultra-short implants, enabling implant placement in situations with limited vertical bone height [2]. In the present study, implant length terminology follows the classification proposed by Al-Johany et al., in which conventional implants are defined as having a length of at least 8 mm, whereas ultra-short implants have a length of 6 mm or less [11]. Although concerns regarding primary stability and biomechanical performance have historically limited their acceptance, extra-short implants have shown favorable clinical outcomes in atrophic ridges, while splinted restorations have been associated with fewer prosthetic complications and lower implant failure rates than non-splinted restorations [12]. In parallel, immediate and early loading protocols have demonstrated favorable clinical outcomes in complete-arch implant rehabilitation [13]. Advances in digital planning and manufacturing have further enabled patient-specific workflows, supported by close coordination between clinical and technical processes [14]. Building on these developments, the present study evaluated a clinical protocol combining multiple ultra-short implants with immediate loading and rigid cross-arch splinting using a patient-specific CAD/CAM inter-implant bar system, aiming to optimize load distribution and limit implant micromotion during early healing while supporting a maxillary overdenture in patients with severe maxillary atrophy.

The aim of this prospective pilot study was to evaluate the clinical feasibility and preliminary outcomes of this treatment concept. We hypothesized that this approach would provide sufficient stability to achieve successful functional rehabilitation in patients with severe maxillary atrophy.

## 2. Materials and Methods

### 2.1. Study Design

This study was designed as a prospective, single-arm pilot study to evaluate the feasibility and early clinical outcomes of a treatment concept using immediately loaded 5.2-mm ultra-short implants connected by a patient-specific CAD/CAM inter-implant bar system (VD CLIC BAR^®^, VD Milling Center, Brugge, Belgium) supporting a maxillary overdenture. Patients were consecutively recruited between 2019 and 2025 at Ghent University Hospital (Belgium) and a private referral clinic (“Het Tandplein”). All surgical and prosthetic treatments were performed at “Het Tandplein”. The study was approved by the Ethics Committee of Ghent University Hospital (Belgium; registration number B670201940813). Written informed consent was obtained from all participants.

### 2.2. Patient Selection

**Figure 1:**
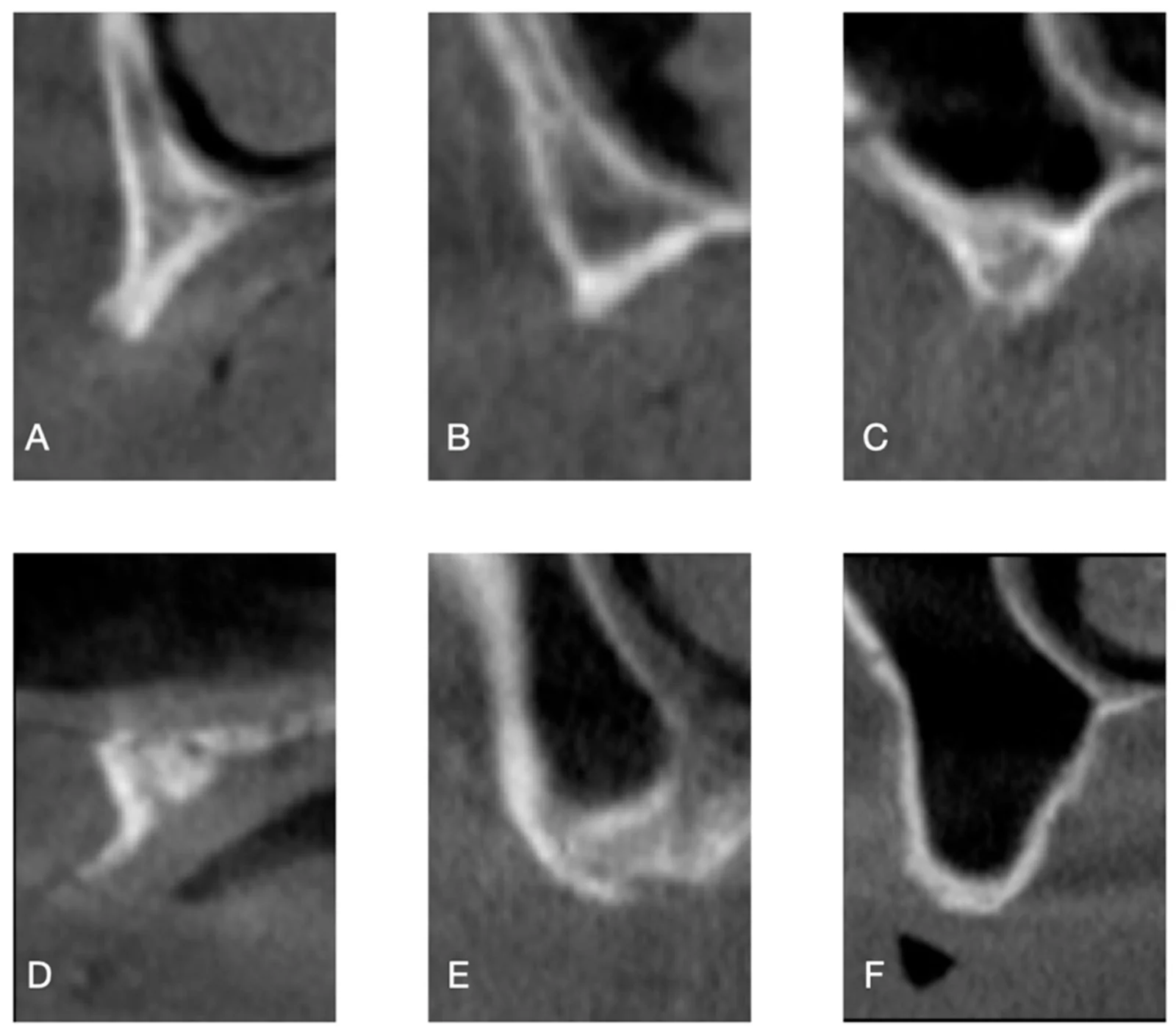
Representative CBCT cross-sectional images illustrating different stages of maxillary ridge resorption according to the Cawood-Howell classification. (**A**–**C**) Cawood-Howell Class V ridge morphology at the lateral incisor, premolar, and molar region respectively. (**D**–**F**) Cawood-Howell Class VI ridge morphology in the corresponding anatomical regions.

Patients were eligible for inclusion if they were completely edentulous in the maxilla and presented with severe maxillary atrophy. Maxillary atrophy was assessed preoperatively using cross-sectional CBCT images. Cross-sectional slices were evaluated at three standardized regions (lateral incisor, first premolar, and first molar). The residual ridge morphology at each site was subsequently classified according to the Cawood-Howell classification, in line with previously described CBCT-based assessment protocols [15]. Patients were eligible when at least 50% of the planned implant sites were classified as Cawood–Howell Class V (flat ridge form with inadequate height and width) or Class VI (depressed ridge form with basal bone loss) [16] (Figure 1).

Exclusion criteria were deliberately limited given the exploratory nature of this pilot study and included uncontrolled systemic disease (ASA IV or higher), medical conditions contraindicating oral surgery, ongoing head and neck radiotherapy or chemotherapy, and active oral infection at the time of surgery. Patients classified as ASA III were not excluded, provided their medical condition was stable and appropriate medical clearance had been obtained. Baseline demographic and clinical data were collected for all included patients and comprised age, sex, ASA classification, smoking status, type of opposing dentition, patient-reported outcomes and implant stability over time.

### 2.3. Preoperative Planning

All patients received an immediately loaded implant-supported maxillary overdenture as a provisional prosthesis following implant placement. All treatments were performed at a private referral clinic (“Het Tandplein”) by the same experienced surgeon (L.V.D.). Patients underwent preoperative clinical and radiographic assessment. Cone-beam computed tomography (Planmeca Promax 3D dental CBCT, Planmeca Oy, Helsinki, Finland) was performed to evaluate residual bone volume. A diagnostic denture set-up was used to assess vertical and horizontal jaw relations and to verify the available prosthetic space. To accommodate the inter-implant bar system, attachment components, denture base material, and prosthetic teeth, a minimum vertical restorative space of approximately 11–15 mm was required [17]. Given the advanced maxillary atrophy in the included patients, sufficient vertical prosthetic space was always available and confirmed during the diagnostic set-up. Implant positions were determined based on a preoperative CBCT planning.

### 2.4. Surgical Procedure

**Figure 2:**
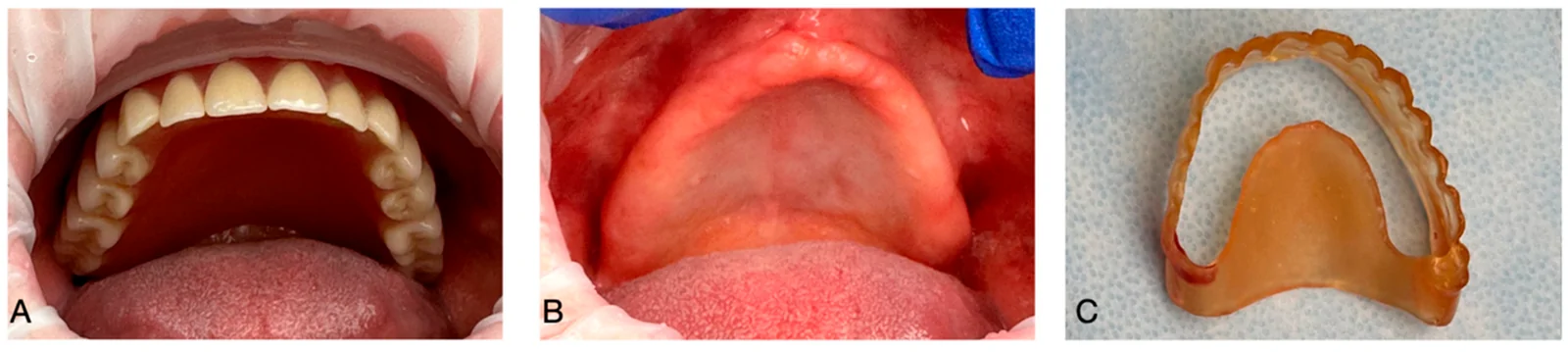
(**A**) Preoperative intraoral view showing the maxillary complete denture. (**B**) Occlusal view of the maxillary ridge, illustrating ridge atrophy. (**C**) Digitally fabricated surgical guide derived from the diagnostic denture set-up, modified with palatal access sleeves to guide implant osteotomies.

Placement was generally aimed at achieving a symmetrical anterior–posterior distribution in the lateral incisor, first premolar, and first molar regions to optimize load distribution and bar support. Following the preoperative assessments, a printed copy of the diagnostic denture was modified with a palatal bur sleeve to serve as a surgical guide (NextDent 5100 3D Printer, Soesterberg, The Netherlands). The surgical guide was used as a prosthetically driven orientation aid to support palatal positioning of the implants and favorable implant angulation, while maintaining a minimum inter-implant distance of approximately 8 mm (Figure 2).

**Figure 3:**
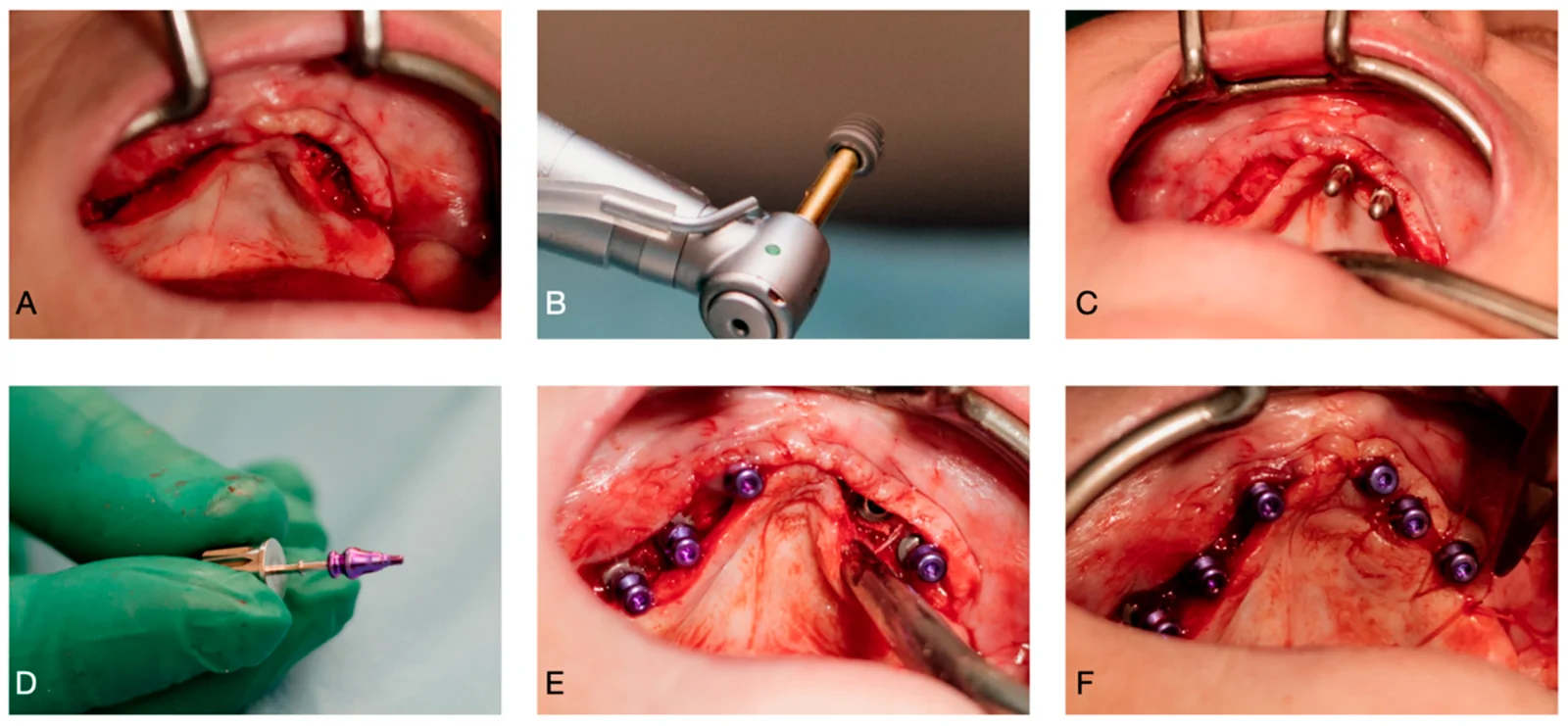
Surgical placement of ultra-short implants. (**A**) Crestal incision and elevation of a full-thickness mucoperiosteal flap. (**B**) CopaSKY ultra-short implant mounted on the implant driver. (**C**) Intraoperative assessment of implant positioning. (**D**–**F**) Placement of CopaSKY TiSi.snap abutment.

All surgical procedures were performed by a single experienced surgeon (LVD) under local anesthesia using 4% articaine hydrochloride and epinephrine 1:100.000 (Septodont, Lancaster, PA, USA). A crestal incision was made along the alveolar ridge, and a full-thickness mucoperiosteal flap was elevated to expose the residual maxillary bone. In the presence of knife-edge ridge morphology, limited alveolar bone reduction was performed to create a flattened implant platform and facilitate osteotomy preparation. Final implant positioning was determined according to prosthetic requirements and available bone volume. Six to eight ultra-short implants (CopaSKY Ultra Short 5.0 × 5.2 mm, Bredent Medical, GmbH & Co.KG, Senden, Germany) were placed according to the manufacturer’s surgical protocol using a low-speed, water-cooled drilling sequence. The final number of implants (6–8) was determined intraoperatively according to the surgeon’s clinical assessment of the available bone volume, bone quality, implant distribution, and achievable primary stability. Implants were positioned slightly subcrestally where possible. Following implant placement, primary implant stability was assessed and retentive titanium abutments (CopaSKY TiSi.snap abutments, COPTISI2, Bredent Medical, Senden, Germany) were connected according to the manufacturer’s recommendations (Figure 3).

Ice packs were provided immediately after surgery. Pain medication (Ibuprofen 600 mg and/or Paracetamol 1 g, maximum three times daily orally) and antibiotics (Amoxicillin 1 g or Clindamycin 300 mg in case of allergy, three times daily for 5 days orally) were routinely prescribed. Detailed written postoperative instructions were discussed thoroughly and given to the patients.

### 2.5. Prosthetic Workflow

**Figure 4:**
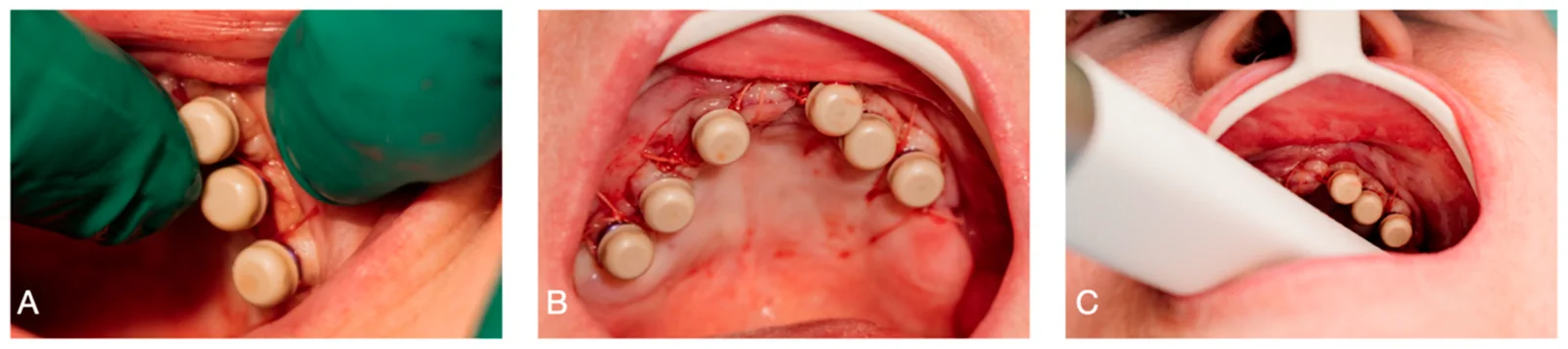
(**A**) Placement of Locator^®^ scan caps onto the TiSi snap abutments following implant insertion. (**B**) Occlusal view showing all scan caps fully seated and aligned prior to digital impression. (**C**) Intraoral scanning using an optical scanner to capture the implant positions and surrounding soft tissues.

Immediate loading was performed using a patient-specific CAD/CAM inter-implant bar system (VD CLIC BAR^®^). Immediately after surgery, Locator^®^ scan caps (Zestdent, Carlsbad, CA, USA) were connected to the retentive titanium TiSi snap abutments and a digital impression was obtained using an intraoral scanner (Trios 3, 3Shape, Copenhagen, Denmark). In addition, the opposing jaw was scanned, and maxillomandibular relations were recorded using the surgical guide as a bite registration aid (Figure 4).

**Figure 5:**
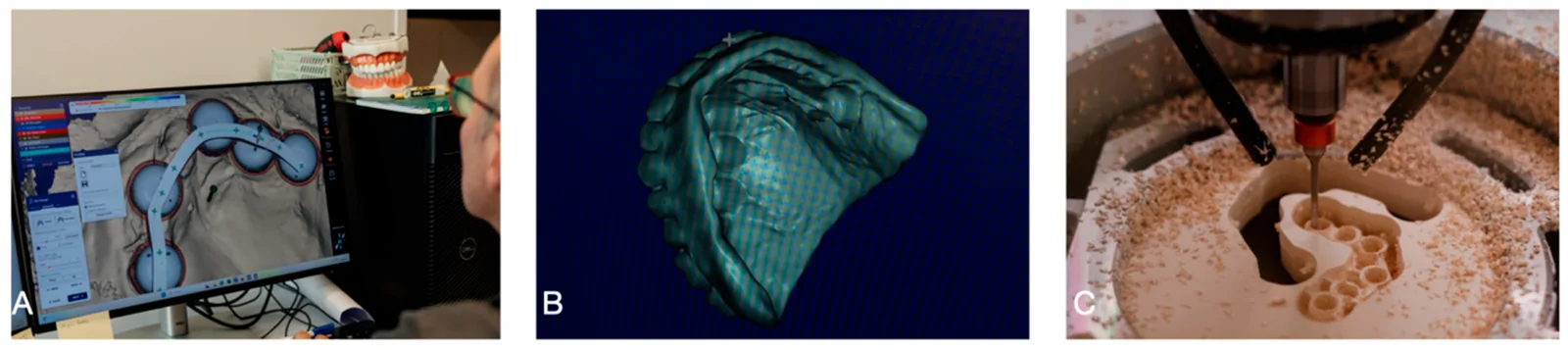
Digital design and fabrication of the immediate implant-retained maxillary overdenture. (**A**) Computer-aided design of the prosthetic framework based on the digital impression. (**B**) Three-dimensional virtual model of the maxillary overdenture. (**C**) CAD/CAM milling of the prosthetic framework (PEEK blank).

The VD CLIC BAR^®^ was designed using Exocad software (DentalCAD, version 2.4 Plovdiv; exocad GmbH, Darmstadt, Germany) and subsequently manufactured from polyetheretherketone (PEEK) (breCAM.BioHPP, Bredent Medical, Senden, Germany) using a CAD/CAM milling machine (DC3, Bredent Medical, Senden, Germany). The bar splinted the implants via a snap-on attachment to the TiSi snap abutments, while the overall framework geometry provided overdenture retention through a friction-based mechanism. In parallel, a telescopic overdenture was designed using the same CAD software (DentalCAD, version 2.4 Plovdiv; exocad GmbH, Darmstadt, Germany) to fit over the inter-implant bar. The denture base was milled from PMMA (breCAM.base, Bredent Medical, Senden, Germany), while the prosthetic teeth were milled separately from high-impact polymer composite (breCAM.HIPC, Bredent Medical, Senden, Germany) using the same milling unit as described above. The denture teeth were segmented into three units (one anterior and two posterior segments) to facilitate precise positioning and adaptation (Figure 5).

Following milling, both the denture base and tooth segments were pretreated with a bonding agent (Visio.link, Bredent Medical, Senden, Germany) and subsequently adhesively luted together, according to the manufacturer’s instructions.

**Figure 6:**
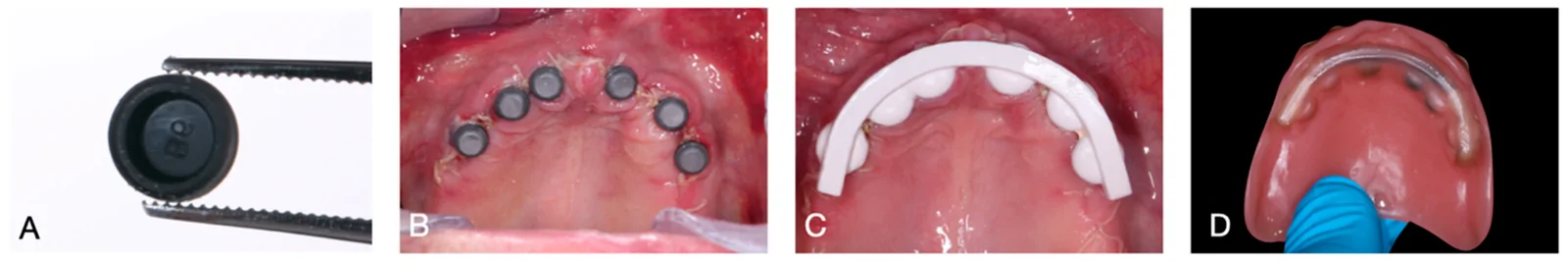
(**A**) Dockloc^®^ denture cap with black laboratory insert. (**B**) Sandblasted Dockloc^®^ denture caps seated on the TiSi snap abutments. (**C**) Patient-specific CAD/CAM inter-implant bar (VD CLIC BAR^®^) after intraoral cementation. (**D**) Telescopic maxillary overdenture.

After completion of the laboratory phase, the prosthesis was delivered intraorally as follows. Compatible Locator^®^ denture caps (Zestdent, Carlsbad, CA, USA) equipped with black laboratory inserts were sandblasted extraorally using 50 µm aluminum oxide (Arseus Lab, Zeist, The Netherlands) and seated passively onto the TiSi snap abutments. The patient-specific CAD/CAM PEEK bar was then positioned intraorally over the denture caps and adhesively luted using DTK-Kleber (Bredent Medical, Senden, Germany). This approach facilitated passive bonding of the bar to the abutments while compensating for minor discrepancies during fabrication of the inter-implant bar. During polymerization, the maxillary overdenture was placed over the bar, and the patient was instructed to close into maximal intercuspation to ensure correct spatial and occlusal relationships during fixation. After complete setting of the adhesive, the prosthesis was removed and the bar was carefully disengaged from the abutments. At this stage, the denture caps were bonded within the PEEK bar. Excess material was meticulously removed using a fine diamond bur and polishing instruments to achieve a smooth and cleansable surface. Finally, occlusion, retention, and soft-tissue adaptation were checked and verified. The laboratory inserts were replaced by red color-coded Dockloc^®^ inserts (Bredent Medical, Senden, Germany), providing approximately 6 N (≈600 g) of manufacturer-specified retention per matrix. This allowed immediate functional loading of the implants through the rigid patient-specific CAD/CAM inter-implant bar while distributing occlusal forces across the splinted implant system. Although the provisional prosthesis was removable, the implants fulfilled the definition of immediate loading, as they were functionally loaded within 48 h of implant placement (Figure 6).

Patients were instructed in daily cleaning of the overdenture, inter-implant bar, and peri-implant tissues using appropriate oral hygiene aids. Follow-up visits during the healing period were scheduled as clinically indicated to assess healing, reinforce oral hygiene, and perform adjustments of the provisional overdenture when necessary.

After a minimum implant integration period of six months, the final prosthetic rehabilitation was executed, including implant-retained overdentures, implant-supported fixed or semi-fixed solutions. The individual choice was based on patients’ needs and expectations, as well as the clinical conditions, such as the available prosthetic space and hygiene accessibility.

### 2.6. Clinical Outcomes

#### 2.6.1. Implant Survival

Primary implant survival was defined as the proportion of initially placed implants that remained in situ and functional throughout follow-up. Reimplantations were recorded separately and excluded from the primary survival analysis. Implant loss was defined as removal of the implant for any reason.

#### 2.6.2. Implant Stability

Implant stability was assessed using the Periotest^®^ device (Gulden Medizintechnik, Bensheim an der Bergstraße, Germany), which quantifies implant stability by measuring the contact time of a tapping rod against the implant-abutment complex and expresses this as a Periotest value (PTV). PTVs range from approximately −8 to +50, with higher values indicating increased implant mobility and lower values reflecting greater stability. After calibration of the device and tightening the abutments according to manufacturer’s recommendations, measurements were performed perpendicular to the transmucosal abutment. The mean of three consecutive measurements per implant was recorded. In line with previous clinical studies, serial Periotest^®^ values were used to monitor changes in implant stability over time rather than to define absolute stability thresholds [18].

#### 2.6.3. Oral Health-Related Quality of Life

Oral Health–Related Quality of Life (OHRQoL) was evaluated using the validated Dutch version of the Oral Health Impact Profile-14 (OHIP-14) questionnaire. This instrument consists of 14 items across seven domains assessing functional limitation, physical pain, psychological discomfort, physical disability, psychological disability, social disability, and handicap. Each item is scored on a 5-point Likert scale (0–4), with higher scores indicating poorer OHRQoL [19]. OHIP-14 scores were recorded preoperatively, early postoperatively, and at the two-year follow-up.

#### 2.6.4. Chewing Ability

Self-reported masticatory function was assessed at the two-year follow-up using a 100-mm visual analogue scale (VAS), reflecting the patient’s perceived ability to chew different types of food, where 0 represented inability to chew and 100 indicated no limitation [20]. Preoperative VAS measurements were not obtained.

### 2.7. Statistical Analysis

All data were analyzed using IBM SPSS Statistics 31.0 (IBM Corp., Armonk, NY, USA). Continuous variables were summarized using mean ± standard deviation or median (interquartile range [IQR]), as appropriate. Categorical variables were summarized using absolute and relative frequencies. Changes in patient-reported outcomes and implant stability over time were analyzed using nonparametric tests, with paired comparisons performed using the Wilcoxon signed-rank test. Repeated implant-level measurements were analyzed using a linear mixed-effects model. Implant survival was analyzed using Kaplan–Meier survival analysis. Data were graphically presented using Kaplan–Meier curves and box-and-whisker plots. Statistical significance was set at *p* < 0.05.

## 3. Results

### 3.1. Population Characteristics

A total of 13 edentulous patients were included (9 females, 4 males). The mean age at surgery was 66.3 ± 10.6 years (range 50–82). Seven patients (53.8%) presented with predominantly Cawood–Howell Class V maxillary atrophy, whereas six patients (46.2%) were classified as Class VI. Baseline demographic and clinical characteristics, including the opposing mandibular dentition, are presented in Table 1.

### 3.2. Clinical Outcomes

#### 3.2.1. Implant Survival

A total of 96 initial implants were placed in 13 patients. Following early implant failures, 8 additional implants were inserted in three patients, resulting in 104 implant placements in total. Implant placement and outcomes according to Cawood–Howell classification are summarized in Table 2.

**Figure 7:**
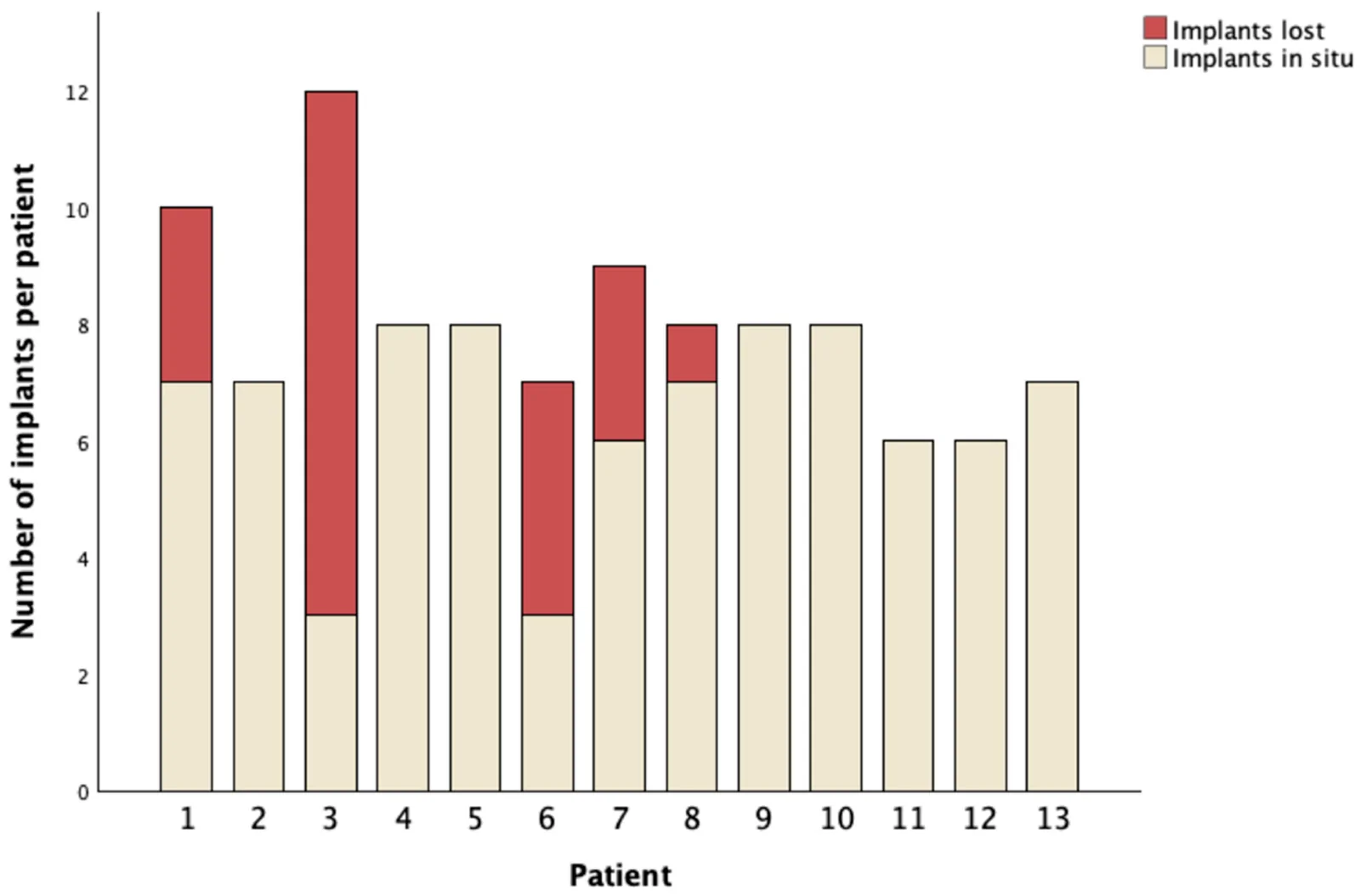
Number of implants placed per patient (including reimplantations), with implants remaining in situ and implants lost indicated separately.

Implant failures occurred predominantly in patients with Cawood–Howell Class VI maxillary atrophy. Of the 16 failures among initially placed implants, 12 occurred in Class VI patients. In addition, all four failures among the reimplanted implants occurred in this group. Implant failures were unevenly distributed across the cohort. The patient-level distribution of implant placement and implant loss is illustrated in Figure 7. Eight of the thirteen patients experienced no implant loss during follow-up, whereas one patient accounted for nine of the twenty implant failures.

**Figure 8:**
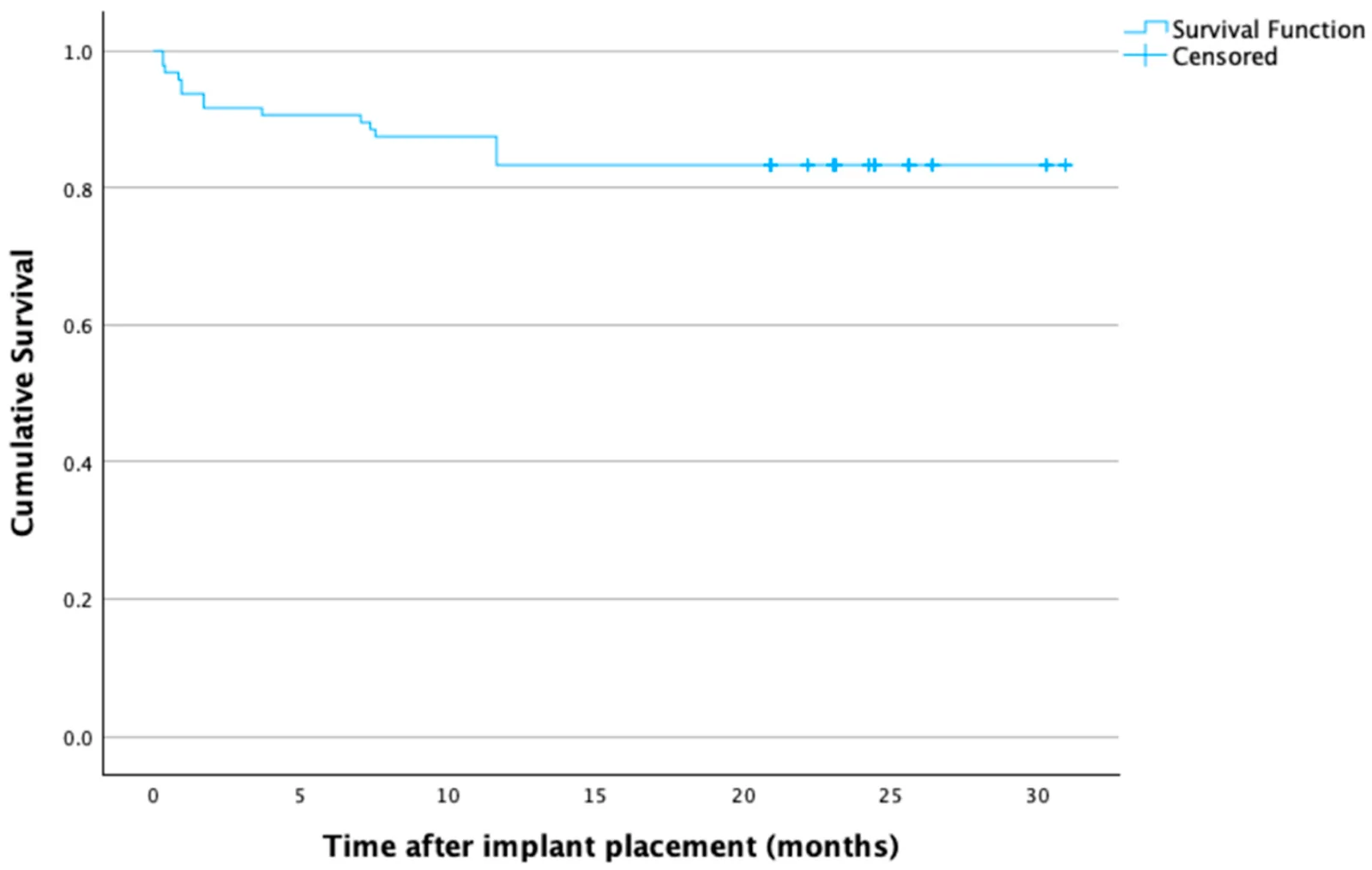
Kaplan–Meier survival curve of initially placed ultra-short implants (excluding reimplantations) up to 30 months of follow-up.

Of the 96 initially placed implants, 16 failed during follow-up, resulting in a primary implant survival rate of 83.3% (95% CI: 75.9–90.7%) after 2 years (Figure 8).

At the final follow-up assessment, 84 implants remained in situ. Of these, 82 were functionally incorporated into the definitive prosthetic rehabilitation, while two implants remained in situ but were not included in the final prosthetic design. Nevertheless, all 13 patients were successfully rehabilitated with an implant-supported prosthesis. Definitive rehabilitation consisted of a TiSi-snap overdenture in seven patients, a bar-retained overdenture in four patients, and a semi-fixed implant-supported prosthesis (Locator Fixed ^®^) in two patients.

#### 3.2.2. Implant Stability

**Figure 9:**
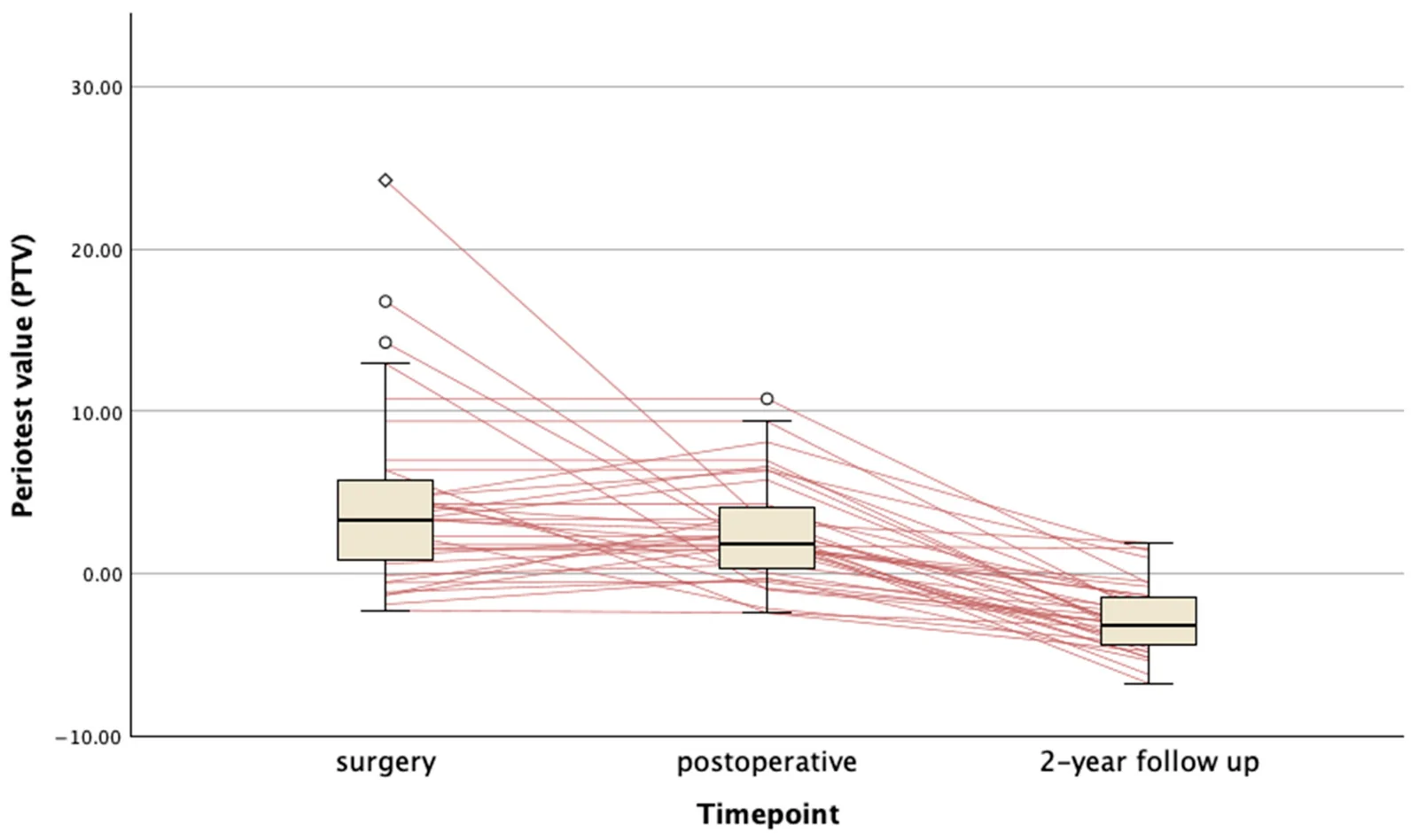
Periotest values (PTV) at surgery, postoperative and 2-year follow-up for implants with complete measurements across all three timepoints (*n* = 35), displayed as boxplots. Individual implant trajectories are shown as red lines connecting repeated measurements from the same implant across the three timepoints. Outliers are indicated by circles and extreme outliers by diamonds.

Implant stability over time was assessed using serial Periotest measurements. Measurements were available for 68 implants at surgery, 87 implants postoperatively (median 20 days after surgery; IQR 12–38 days), and 62 implants at approximately 2 years of follow-up. Complete measurements across all three timepoints were available for 35 implants. Paired surgery–2-year data were available for 40 implants, and paired surgery–postoperative data for 55 implants. In implants with paired surgery and 2-year measurements (*n* = 40), median Periotest values decreased significantly from 3.50 (IQR 1.20–8.77) at surgery to −3.22 (IQR −4.30–−1.37) at 2-year follow-up (Wilcoxon signed-rank test, *p* < 0.001), indicating increased implant stability over time. No significant difference was observed between surgery and the postoperative assessment (*n* = 55, Wilcoxon signed-rank test, *p* = 0.891). A linear mixed-effects model with timepoint as a fixed effect and patient as a random intercept demonstrated a significant effect of time on Periotest values (F(2, 211) = 42.4, *p* < 0.001), confirming a progressive increase in implant stability over time after accounting for clustering within patients (Figure 9).

#### 3.2.3. Oral Health-Related Quality of Life

**Figure 10:**
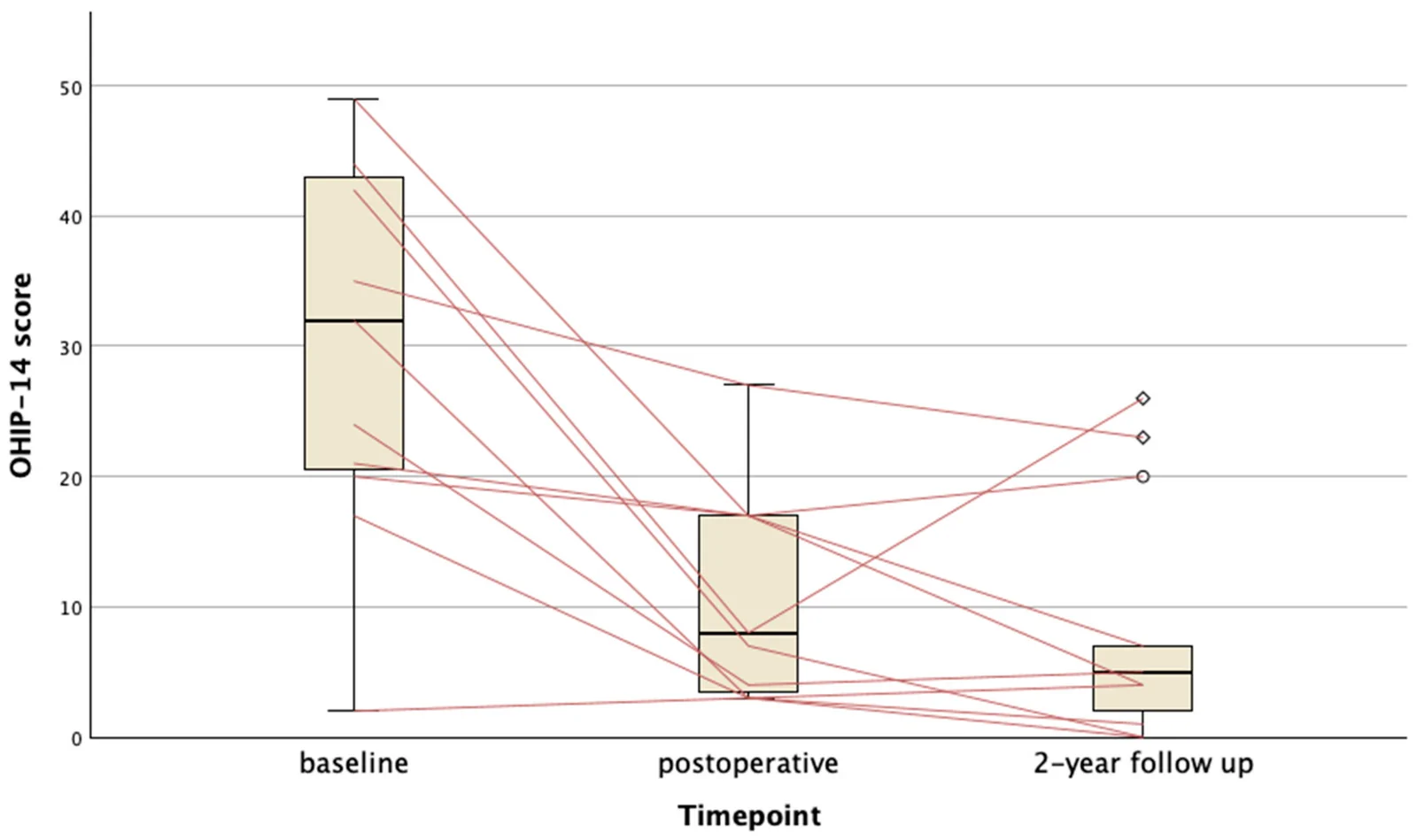
OHIP-14 scores at baseline (*n* = 13), postoperative (*n* = 11), and 2-year follow-up (*n* = 11), displayed as boxplots. Individual patient trajectories are shown as red lines connecting repeated measurements from patients with complete data across all three timepoints (*n* = 10), illustrating a progressive decrease in OHIP-14 scores over time. Outliers are indicated with circles and extreme outliers with diamonds. Three patients with persistently elevated scores at 2-year follow-up are visible as outliers.

Oral health–related quality of life improved significantly over time and median OHIP-14 scores decreased from 32.0 (IQR 20.0–44.0) preoperatively to 4.0 (IQR 1.0–20.0) after 2 years (Wilcoxon signed-rank test, Z = −2.76, *p* = 0.006). A significant improvement was already observed immediately after surgery, with median OHIP-14 scores decreasing from 28.0 (IQR 20.0–42.0) at baseline to 7.5 (IQR 3.0–17.0) (*n* = 10) (Wilcoxon signed-rank test, Z = −2.70, *p* = 0.007). No significant further change was observed between the postoperative assessment and 2-year follow-up (*n* = 11, Z = −1.291, *p* = 0.197) (Figure 10).

#### 3.2.4. Chewing Ability 

Patient-reported chewing ability was assessed at the 2-year follow-up in all 13 patients. Overall, patients reported favorable functional outcomes, with a median VAS score of 82.7 (IQR 50.1–91.9; range 27.8–96.7), and the majority of patients indicating good to excellent chewing ability.

## 4. Discussion

This prospective pilot study evaluated the feasibility of a treatment concept using immediately loaded 5.2-mm ultra-short implants connected by a patient-specific CAD/CAM inter-implant bar system (VD CLIC BAR^®^) to support a maxillary overdenture in patients with severe maxillary atrophy. The primary implant survival rate was 83.3% after two years. Implant failures occurred predominantly during the early healing phase, before definitive prosthetic rehabilitation. Based on the clinical findings at implant removal, these failures were considered most consistent with lack of osseointegration rather than peri-implant infection or late biomechanical overload. No additional implant losses were observed after delivery of the definitive prosthetic rehabilitation during the available follow-up period. Implant failures were not evenly distributed across the cohort but were clustered within a limited number of patients, with nine implant losses occurring in a single patient. Although failures occurred in both Cawood–Howell Class V and Class VI maxillae, they were more frequent in the latter. While this distribution may suggest that anatomical factors, such as the amount of residual alveolar bone, could influence treatment outcomes, the clustering of failures also indicates that patient-related factors may have contributed. As this pilot study was not designed or powered to investigate these associations, no conclusions regarding predictors of implant failure can be drawn. Early implant failures were managed by placement of additional 5.2-mm ultra-short implants, allowing definitive implant-supported rehabilitation in all patients. This observation suggests that early implant loss did not necessarily compromise the overall treatment outcome, although additional surgical intervention was required. Importantly, none of the patients required immediate conversion to more complex reconstructive procedures.

While the clinical performance of extra-short dental implants has been extensively investigated for single-tooth and partial fixed restorations, evidence supporting their use in fully edentulous patients remains limited. Available data on extra-short implants in full arch rehabilitation are largely confined to the mandible [12,21,22] or to maxillary overdentures in which extra-short implants were combined with standard-length implants rather than used as the sole means of support [23]. To our knowledge, this is the first clinical study describing an immediately loaded maxillary overdenture supported exclusively by 5.2-mm ultra-short implants in patients with severe maxillary atrophy. Given the limited evidence on the use of ultra- or extra-short implants in complete arch rehabilitations, comparisons with available overdenture and full-arch studies should therefore be interpreted with caution. Guljé et al. reported on mandibular overdentures supported by four 6-mm implants with a 1-year follow-up, demonstrating a high implant survival rate of 96% in a cohort of 12 patients [24]. In a prospective cohort study, Ewers et al. evaluated fixed fiber-reinforced composite full-arch prostheses supported by four 5.0-mm ultra-short implants in severely atrophic mandibles, reporting an implant survival rate of 97.2% with stable marginal bone levels over a follow-up period of up to eight years [25]. In a systematic review and meta-analysis, Moraschini et al. compared extra-short 4-mm implants with longer implants placed in the mandible and found no significant differences in implant survival after one year of function, while marginal bone loss and biological complications were significantly lower for extra-short implants [22]. Similarly, Ravidà et al. reported an overall survival rate of approximately 94% of extra-short dental implants, with higher survival observed in the mandible compared to the maxilla. Notably, restorations supported by splinted implants have been associated with more favorable clinical outcomes than non-splinted configurations [12].

In the present study, immediate loading was facilitated by rigid cross-arch splinting, which may have enhanced primary stability and load distribution during early healing. This may be particularly relevant for ultra-short implants, where minimizing micromotion is considered important for successful osseointegration. Finite element analyses have shown that occlusal stresses are concentrated in the crestal cortical bone, making implant diameter more influential than implant length for peri-implant stress distribution [26,27]. Although no histological data were available in the present cohort, the biological rationale for the use of 5.2-mm ultra-short implants deserves consideration. Concerns regarding reduced implant length have traditionally focused on the assumption that a smaller bone-to-implant contact (BIC) area compromises load-bearing capacity. However, available histological evidence suggests that successful osseointegration depends not only on the absolute BIC surface area, but also on the quality of the bone–implant interface and functional loading conditions [28]. Together, these biomechanical and biological considerations provide the theoretical rationale for combining multiple ultra-short implants with rigid cross-arch splinting in immediately loaded rehabilitations.

The CAD/CAM-milled BioHPP bar was selected because this polymer-based CAD/CAM material possesses favorable biomechanical characteristics, including low weight, a substantially lower elastic modulus than titanium, and compatibility with fully digital CAD/CAM workflows. BioHPP has also been applied successfully in bar-retained implant overdentures, although long-term clinical evidence remains limited [29]. In the present protocol, the overdenture was designed to engage the bar directly, without additional clips. This retention concept requires a relatively large contact surface between the bar and the intaglio surface of the overdenture. These design considerations were based on protocol development and were not formally evaluated in the present study.

Patient-reported oral health–related quality of life improved significantly following prosthetic loading, with a reduction in OHIP-14 scores that was maintained throughout follow-up. Self-reported chewing ability at the 2-year follow-up also indicated favorable functional outcomes. Implant stability, assessed using serial Periotest measurements, improved over time, reflecting increased mechanical stability of surviving implants at follow-up [18]. In the present study, the immediate overdenture primarily served as a provisional loading strategy to enable early functional rehabilitation and implant splinting. Definitive prosthetic designs were individualized based on patient-specific factors. Therefore, the favorable patient-reported outcomes observed in this cohort likely reflect the overall treatment concept rather than a specific prosthetic modality.

Additional perspective can be drawn from the literature on mini dental implants (MDIs) used to support maxillary overdentures in patients with severe alveolar atrophy. In a systematic review and meta-analysis, Vi et al. reported an overall implant survival of approximately 77% for MDIs retaining maxillary overdentures, with implant losses occurring predominantly during the early follow-up period [30]. Despite this lower implant survival, patient-reported outcomes demonstrated significant improvements in oral health–related quality of life, denture stability, and chewing ability, and prosthesis survival remained high. These observations are further supported by a recent, long-term prospective cohort study. Van Doorne et al. evaluated maxillary overdentures supported by 5–6 flaplessly placed MDIs [31]. Although a proportion of implants were lost over time, most overdentures remained functional, peri-implant conditions of the remaining implants were favorable, and improvements in patient-reported outcomes were maintained throughout follow-up. Together, these findings suggest that in anatomically compromised maxillae, higher implant loss rates may coexist with stable prosthetic function and favorable patient-reported outcomes.

Limitations of this study include the small sample size and the absence of a control group, which restrict direct comparison with other treatment approaches. The two-year follow-up provides insight into early and mid-term outcomes but does not capture long-term implant and prosthetic performance. The heterogeneity of the patient cohort may have contributed to variability in outcomes. Furthermore, biological and prosthetic outcomes—including marginal bone loss, peri-implant tissue health, prosthetic complications, maintenance procedures, and prosthetic adjustments—were not systematically recorded, limiting the comprehensive evaluation of long-term implant and prosthetic performance. The prosthetic protocol also combined components originating from different manufacturers. Although this pragmatic approach enabled development of the proposed treatment concept and performed satisfactorily in this pilot study, its off-label nature may limit reproducibility. Finally, all treatments were performed by a single experienced surgeon in a specialized referral center. The reproducibility and generalizability of this treatment concept to routine clinical practice remain to be established.

## 5. Conclusions

This prospective pilot study introduces a graft-avoiding treatment concept based on immediately loaded 5.2-mm ultra-short implants splinted with a patient-specific CAD/CAM inter-implant bar system (VD CLIC BAR^®^) to support a maxillary overdenture in carefully selected patients with severe maxillary atrophy. The present findings suggest that this protocol is clinically feasible and may represent an additional treatment option for selected patients in whom conventional bone augmentation procedures are contraindicated, declined, and where more remote-anchorage concepts would otherwise be considered. However, given the pilot design, limited sample size and absence of a control group these findings should be regarded as preliminary. Further prospective controlled studies with larger cohorts, appropriate comparison groups, and longer follow-up are required to determine the long-term predictability and clinical applicability of this treatment approach.

## Figures and Tables

**Table 1:** Baseline characteristics of the study population.

Characteristic	*n*	%
Patients	13	
Age at surgery, mean ± SD (years)	66.3 ± 10.6	
Female	9	69.2
Male	4	30.8
Smokers	2	15.4
**Cawood–Howell classification**		
Class V	7	53.8
Class VI	6	46.2
**Initial implants placed per patient**		
6 implants	2	15.4
7 implants	4	30.8
8 implants	7	53.8
**Opposing dentition**		
Natural dentition	6	46.2
Natural dentition + RPD	2	15.4
Implant-retained overdenture	3	23.1
Implant-supported fixed prosthesis	2	15.4

**Table 2:** Implant placement and outcomes according to Cawood–Howell classification.

	Class V	Class VI	Total
Initial implants, *n*	51	45	96
Initial implant failures, *n*	4	12	16
**Initial implant failure rate, %**	7.8	26.7	16.7
Reimplantations, *n*	2	6	8
Reimplantations failures, *n*	0	4	4
Total implants placed, *n*	53	51	104
Total implant failures, *n*	4	16	20
**Total implant failure rate, %**	7.5	31.4	19.2

## Data Availability

The data presented in this study are available on request from the corresponding author, as they are not publicly available due to privacy or ethical restrictions.
